# Uncovering the gene variants in a global cohort of patients with unexplained increased left ventricular wall thickness using next-generation sequencing

**DOI:** 10.1186/s12872-026-05834-5

**Published:** 2026-04-17

**Authors:** Michael Arad, Andrea Virginia Ferreira Chaves, Murillo Antunes, Abeer Bakhsh, Kenneth I. Berger, Tse Hung Fat, Armando Alves da Fonseca, Adriana Furtado, Irina Maksimova, Sandra Marques e Silva, Manish Maski, Enrique Monjes, Nelson E. Murillo Benitez, Eduardo Ortuño Campos, Márcia Gonçalves Ribeiro, Maria Juliana Rodriguez-González, Wen-Chung Yu, Huseyin Onay

**Affiliations:** 1 Cardiomyopathy Clinic and Heart Failure Institute, Leviev Heart Center, Sheba Medical Center, Sackler School of Medicine, Tel Aviv University, Tel Aviv, Israel; 2 Department of Cardiology, Hospital Agamenon Magalhães, Recife, Brazil; 3 RARUS, Referral Service for Rare Disease, Recife, Brazil; 4 Medicine Department, Mauricio de Nassau University Center UNINASSAU, Recife, Brazil; 5 The Heart Institute, InCor, University of São Paulo Medical School, São Paulo, Brazil; 6 Department of Cardiology, São Francisco University (USF), Bragança Paulista, São Paulo, Brazil; 7 King Abdullah Medical City, Makkah, Saudi Arabia; 8 Sanofi, Global Medical Affairs-Rare Disease, Cambridge, USA; 9 Department of Medicine, Queen Mary Hospital, School of Clinical Medicine, LKS Faculty of Medicine, The University of Hong Kong, Pok Fu Lam, Hong Kong; 10 DLE Laboratory/ Fleury S.A, Rio de Janeiro, Brazil; 11 GEDORAC/DCC/SBC–Study Group on Rare Diseases with Cardiac Impairment from Brazilian Society of Cardiology, Hospital de Base do Distrito Federal (HBDF), Brasilia, Brazil; 12 Vertex Pharmaceuticals, Incorporated, Boston, USA; 13 Department of Cardiac Electrophysiology, HIGA San Martin de La Plata, Buenos Aires, Argentina; 14 Santiago of Cali University and Clinical of Occidente, Cali, Colombia; 15 Colombian Society of Cardiology and cardiovascular surgery, Cardioprevent IPS, Cali, Colombia; 16 Hospital Universitario Austral, Buenos Aires, Argentina; 17 Department of Pediatrics, Faculty of Medicine, Federal University of Rio de Janeiro, Rio de Janeiro, Brazil; 18 Department of Heart Failure, Cardioinfantil Foundation, Cardiac Institute, Bogota, Colombia; 19 Cardiovascular Center, Taipei Veterans Hospital and National Yang Ming Chiao Tung University, Taipei, Taiwan; 20 Multigen Genetic Diseases Diagnosis Center, Mansuroglu Mah, Bayraklı, Izmir Turkey

**Keywords:** Next-generation sequencing, Left ventricular wall thickness, Hypertrophic cardiomyopathy, Transthyretin cardiac amyloidosis, Fabry disease, Alpha-galactosidase A

## Abstract

**Background:**

Genetic analysis using massive parallel sequencing is crucial for the accurate and early diagnosis of hereditary hypertrophic cardiomyopathies and their phenocopies, especially transthyretin cardiac amyloidosis (ATTR-CA) and Fabry disease (FD). This study extends the cardio next-generation sequencing (NGS) pilot study by investigating the detection rate of gene variants causing increased left ventricular wall thickness (LVWT) using an expanded 19-gene NGS panel in a larger global cohort.

**Methods:**

This study included 2068 patients with unexplained increased LVWT enrolled at cardiological clinics across 22 countries/regions between 2020 and 2022. The NGS panel comprised 19 genes associated with hypertrophic cardiomyopathy (HCM) and its phenocopies. Sequencing was performed using the Illumina NextSeq 500 and NovaSeq 6000 systems, with variant interpretation performed according to the American College of Medical Genetics and Genomics guidelines. Novel variants were analyzed using the Human Gene Mutation Database (HGMD^®^), Franklin, and VarSome.

**Results:**

Among the 2068 patients, 453 patients were positive for pathogenic/likely pathogenic variants (21.9%). The diagnostic yield for HCM was 18.4%, while that of HCM phenocopies was 3.5%, including ATTR-CA (1.5%), and FD (0.9%). In patients with a positive test (HCM or HCM phenocopies), the most prevalent HCM-related variants were *MYBPC3* and *MYH7* (36.4% and 34.4% of all positive samples, respectively), whereas *TTR* (7.1%) and *GLA* (4.0%) were the most common phenocopy variants. Other classical phenocopies, Noonan syndrome, Danon disease, and *PRKAG2*, comprised another 2.0%, 0.9%, and 0.7% of the cohort, respectively. The mean ages for patients with HCM sarcomeric gene variants, HCM phenocopy variants, FD, and ATTR-CA were 45.1 ± 17.6, 50.9 ± 23.7, 51.1 ± 19.4, and 64.6 ± 19.0 years, respectively.

**Conclusion:**

This study demonstrates the need to include *GLA* and *TTR* in NGS panels for patients with increased unexplained LVWT. NGS effectively identifies phenocopies often missed by imaging. Using a large, diverse cohort, this study reveals the prevalence of FD and ATTR-CA in patients with unexplained increased LVWT, reinforcing the importance of NGS for early diagnosis and targeted therapy.

**Supplementary Information:**

The online version contains supplementary material available at 10.1186/s12872-026-05834-5.

## Background

Next-generation sequencing (NGS) is an analytical method for genetic testing that significantly advances our ability to determine genetic diseases in patients and identify relatives at risk of inheriting these conditions [1, 2]. Using it enables timely treatment interventions or preventive measures, which are crucial for managing inherited disorders. NGS stands out as one of the essential diagnostic tools for the early diagnosis of genetic disorders. Its widespread availability across a range of countries makes NGS a robust platform for identifying variants associated with various genetic conditions [3]. Along these lines, NGS can serve as a good platform for faster identification of variants associated with hypertrophic cardiomyopathy (HCM) and its phenocopies, such as Fabry disease (FD) and transthyretin cardiac amyloidosis (ATTR-CA) [4, 5]. ATTR-CA is an autosomal dominant disorder caused by pathogenic variants in the TTR gene, which lead to destabilization of the transthyretin tetramer and deposition of amyloid fibrils in tissues, including the myocardium. Over 130 TTR variants have been described, with Val30Met, Val122Ile, and Glu89Gln being the most common worldwide [6]. FD is an X-linked lysosomal storage disorder caused by variants in the *GLA* gene, resulting in deficient α-galactosidase A activity and progressive accumulation of globotriaosylceramide (Gb3) in multiple organs, including the heart [7]. Both conditions may present with increased left ventricular wall thickness (LVWT) mimicking HCM, leading to under-recognition and delayed diagnosis if genetic testing is not performed [8]. However, data on the real-world implementation of multi-gene NGS panels across diverse geographic regions remain limited.

Reliable diagnosis of HCM, ATTR-CA, and FD is vital for selecting effective treatment and prevention strategies [9, 10]. It also facilitates the initiation of clinical and genetic surveillance and counseling of family members [11]. Using NGS diagnostics can enhance early diagnosis, increase the recognition of pathogenic variants causing genetic diseases, and improve clinical care. This is particularly important for conditions such as FD and ATTR-CA, for which targeted therapies are available, making early and accurate detection crucial for leveraging maximal benefits from existing treatment and improving patient outcomes [12, 13]. While most individuals with HCM carry a single pathogenic variant in sarcomeric genes such as *MYH7* or *MYBPC3*, individuals harboring multiple variants have also been identified, which may be associated with earlier onset or more severe phenotypes [14]. However, the frequency and clinical significance of these double variants remain incompletely understood, particularly in diverse or understudied populations. Differential diagnosis is critical when dealing with unexplained increased LVWT, as it can be indicative of various underlying conditions [9, 11]. The early distinction of HCM phenocopies is essential because they have distinct prognostic implications, treatment strategies, and disease progression compared to sarcomeric HCM [15]. Enzyme replacement therapy (ERT) and pharmacological chaperone therapy for FD and disease-modifying agents for ATTR-CA can improve patient outcomes, underscoring the importance of precise and early genetic diagnosis [9, 16, 17].

This study extends the cardio NGS pilot study, which used a 17-gene NGS panel to investigate the detection rate of variants related to common forms of HCM and its phenocopies in patients with unexplained increased LVWT from six countries: Mexico, Colombia, Turkey, Brazil, Israel, and Saudi Arabia [18]. The pilot study revealed that the most prevalent pathogenic gene variants associated with HCM were *MYH7* (11.2%) and *MYBPC3* (7.5%), while the most prevalent HCM phenocopy was ATTR-CA (1.1%). The diagnostic yield for FD was 0.6%, and the overall diagnostic yield for genetically confirmed HCM and HCM phenocopies (pathogenic/likely pathogenic variants) was 24.5%.

Building on the protocol of the previous cardio NGS pilot study, the current study incorporated a few significant changes [18]. Two additional genes, *CSRP3* and *JPH2*, were added to the previously used 17-gene panel, enhancing its utility as a genetic diagnostic tool for cardiologists. The study population was expanded to include 2068 patients from 22 countries/regions, and the inclusion criteria were broadened to an increased LVWT of ≥ 12 mm instead of ≥ 13 mm, allowing the inclusion of patients at earlier stages of cardiac involvement. Furthermore, as part of the diagnostic process, the α-galactosidase A (α-Gal A) enzyme activity and globotriaosylsphingosine (lyso-Gb3) levels in patients diagnosed with *GLA* variants were also investigated, providing an additional step to assess variants’ pathogenicity. This study aims to improve understanding of the genetics of HCM and its phenocopies by analyzing a large cohort and incorporating advanced diagnostic tools to reveal the prevalence and distribution of pathogenic variants.

## Materials and methods

### Sample population

This study is a multicentric analysis of 2068 patients (index cases of different families) from 22 countries/regions with unexplained increased LVWT. The 22 countries/regions include the Republic of Colombia (Colombia), the Federative Republic of Brazil (Brazil), the Argentine Republic (Argentina), the People’s Democratic Republic of Algeria (Algeria), the State of Israel (Israel), the Republic of Kazakhstan (Kazakhstan), the United Mexican States (Mexico), Hong Kong (Special Administrative Region, People’s Republic of China), the Republic of Peru (Peru), Taiwan (Republic of China), the Kingdom of Saudi Arabia (KSA), the Republic of Türkiye (Turkey), United Arab Emirates (UAE), the Republic of Panama (Panama), the Republic of Chile (Chile), the Republic of Guatemala (Guatemala), the Republic of Costa Rica (Costa Rica), the Republic of South Africa (South Africa), the Republic of El Salvador (El Salvador), Dominican Republic, the Republic of Ecuador (Ecuador), and the State of Kuwait (Kuwait). Patients were recruited from a broad network of general cardiology clinics and multidisciplinary cardiovascular services participating in the testing program. In these real-world settings, genetic testing is ordered for patients with unexplained increased LVWT of any suspected etiology, rather than exclusively for individuals with a high pre-test probability of HCM.

### Inclusion criteria

The study included individuals with LVWT of 12 mm or greater in at least one segment of the left ventricular myocardium, as assessed using imaging techniques such as cardiac magnetic resonance imaging or echocardiography, provided no conditions were causing abnormal cardiac loading.

### Exclusion criteria

Patients were excluded from the study if they exhibited increased LVWT caused by severe hypertension, defined as a systolic blood pressure of ≥ 180 mmHg and/or a diastolic blood pressure of ≥ 120 mmHg. Additional exclusion criteria included severe aortic stenosis, characterized by an aortic valve area of ≤ 1 cm², or a genetically confirmed diagnosis of HCM or its mimicking conditions.

### Ethics approval and patient consent to participate

The authors are accountable for all aspects of the work and ensure that questions related to the accuracy or integrity of any part of the work are appropriately investigated and resolved. This study adhered to the ethical guidelines outlined in the Declaration of Helsinki. Signed informed consent forms were obtained from all patients for blood sampling and analysis, followed by approval by the Institutional Review Board. The study received approval from the Instituto de Puericultura e Pediatria Martagão Gesteira/Federal University of Rio de Janeiro under approval no. CAAE 53630421.3.0000.5264.

### Procedures

The blood samples were collected between 2020 and 2022, without identifiable information about the patients, as peripheral dried blood spot (DBS) samples on filter paper. Demographic data, including age, sex, and geographic origin, were recorded simultaneously with sample collection. Samples were analyzed at the Specialized Laboratorial Diagnosis (DLE: Diagnósticos Laboratoriais Especializados) in São Paulo, Brazil.

The selection of the NGS panel Table 1 was based on national and regional epidemiology, global prevalence data, and local technical capabilities. The gene panel was designed to include genes with strong or definitive evidence for association with HCM and its phenocopies, in accordance with current evidence and clinical guidelines. This targeted approach prioritized clinical utility and cost-effectiveness while minimizing the detection of variants of uncertain significance (VUS), which can complicate variant interpretation and genetic counseling. The guidelines from the American College of Medical Genetics and Genomics were used to classify the pathogenicity of the identified variants [19]. These guidelines categorize variants into five classes: pathogenic, likely pathogenic (> 90% probability of being pathogenic), uncertain significance, likely benign (> 90% probability of being benign), and benign [19]. The gene panel was developed to identify single nucleotide variants and small insertions/deletions (indels; nearly 15–25 base pairs) present in the coding sequences of DNA. This panel also helps detect known splice regions and flanking regions (20 bp adjacent to each exon) of the targeted genes using DBS samples.

**Table 1:** 19-gene panel to detect mutations causing HCM and HCM phenocopies in unexplained increased LVWT

Sl. No.	Gene	Protein	Locus	Exons sequenced	OMIM	HCM/HCM phenocopy
1	*MYBPC3*	Myosin-binding protein C3	11p11.2	35	600,958	HCM
2	*MYH7*	Myosin heavy chain 7 or beta	14q11.2	40	160,760	HCM
3	*TNNI3*	Cardiac troponin I cardiac type	19q13.42	8	191,044	HCM
4	*TNNT2*	Cardiac troponin T cardiac type	1q32.1	17	191,045	HCM
5	*TPM1*	Alpha tropomyosin	15q22.2	14	191,010	HCM
6	*MYL3*	Myosin light chain 3	3p21.31	7	160,790	HCM
7	*MYL2*	Myosin light chain 2	12q24.11	7	160,781	HCM
8	*ACTC1*	Cardiac alpha-actin	15q14	7	102,540	HCM
9	*CSRP3*	Cysteine- and glycine-rich protein 3	11p15.1	6	600,824	HCM
10	*JPH2*	Junctophilin-2	20q13.12	5	605,267	HCM
11	*TNNC1*	Troponin C1, slow skeletal and cardiac type	3p21.1	6	191,040	HCM
12	DES*	Desmin	2q35	9	125,660	HCM phenocopy
13	FLNC*	Filamin C	7q32.1	48	102,565	HCM phenocopy
14	*GLA*	α-galactosidase A	Xq22.1	7	300,644	HCM phenocopy
15	*LAMP2*	Lysosomal-associated membrane protein 2	Xq24	10	309,060	HCM phenocopy
16	*PLN*	Phospholamban	6q22.31	2	172,405	HCM phenocopy
17	*PRKAG2*	AMP-activated protein kinase subunit-γ-2	7q36.1	22	602,743	HCM phenocopy
18	*PTPN11*	Protein tyrosine phosphatase non-receptor type 11	12q24.13	16	176,876	HCM phenocopy
19	*TTR*	Transthyretin	18q12.1	4	176,300	HCM phenocopy

*Although *DES* is not a typical HCM phenocopy gene, it can present with HCM-like phenotypes in desmin-related myopathies [20]. Similarly, *FLNC* mutations causing myofibrillar myopathy (MFM5; OMIM #609524) may manifest with HCM-like cardiac hypertrophy; therefore, both were included here as phenotypic mimickers

*HCM* Hypertrophic cardiomyopathy, *LVWT* Left ventricular wall thickness

Genomic DNA was extracted and prepared for sequencing. The preparation of genomic libraries was conducted using the Agilent SureSelect XT HS and XT Low Input Custom protocols. Sequencing was performed on Illumina NextSeq 500 and NovaSeq 6000 systems to achieve high data coverage, with comprehensive quality control procedures ensuring sample integrity. Sequencing data were processed using a validated bioinformatics pipeline designed to align reads and identify genomic variants. The alignment was performed against the human reference genome (GRCh37/hg19) using an in-house tool (‘DLE-Tool’), which integrates the Burrows-Wheeler Aligner (v0.7.15) for mapping. Variant calling for both single-nucleotide variants and insertions/deletions was based on mpileup, and functional annotation was conducted using the Ensembl Variant Effect Predictor (VEP, v105), enabling the assessment of variant pathogenicity and classification as pathogenic, likely pathogenic, or of uncertain significance (VUS). For variants of uncertain or novel nature, additional interpretation was conducted using the Franklin platform by Genoox. The Human Gene Mutation Database (HGMD^®^) was utilized to verify the novelty of *GLA* variants, while further classification of novel alterations was supported by the Franklin and VarSome databases. Detailed protocols and procedures are described in the previous publication by Silva et al. [18].

### Measurements of α-Gal A enzyme activity and lyso-Gb3 concentrations

The enzyme activities of α-Gal A and lyso-Gb3 concentrations were determined using the DBS samples. α-Gal A enzyme activity was measured via the flow injection analysis tandem mass spectrometry (FIA-MS/MS) method using the Revvity LSD kit, operating in multiple reaction monitoring (MRM) mode. The established reference values for α-Gal A enzyme activity were as follows: for newborns up to 30 days, normal activity was defined as ≥ 2.57 µmol/L/h, while for infants and adults, normal activity was ≥ 1.68 µmol/L/h. The limit of detection for this method was 0.97 µmol/L/h.

Lyso-Gb3 concentrations were ascertained with the use of a validated liquid chromatography-tandem mass spectrometry (LC-MS/MS) quantitative analysis developed as an in-house method, also operating in MRM mode. For lyso-Gb3, the normal reference value was set at ≤ 0.8 ng/mL, with a detection limit of 0.2 ng/mL.

## Results

A total of 2068 samples were analyzed between 2020 and 2022. The majority of the patients were males (1282 [62%]), and 783 [37.9%] were females; the sexes of 3 [0.1%] individuals were not recorded (Table 2). Among the 2068 patients, 453 patients were positive for pathogenic/likely pathogenic variants (21.9%), among which three patients had double variants. Of the total 453 positive cases, 380 were associated with classical HCM genes (18.4%), while 73 involved HCM phenocopy-related genes (3.5%), including *TTR* and *GLA*. Eighteen (0.9%) patients were diagnosed with FD (11 [61.1%] males, 7 [38.9%] females). The mean age of the patients with HCM and HCM phenocopy variants was 46.1 ± 18.7 years, whereas those with confirmed FD had a mean age of 51.1 ± 19.4 years. The diagnostic yield of ATTR-CA was 1.5% ([*N* = 32]; 21 [65.6%] males, 10 [31.3%] females, and the sex of 1 [3.1%] individual was not recorded). The mean age of the patients with ATTR-CA was 64.6 ± 19.0 years (Table 2).

**Table 2:** Demographics of patients with HCM, HCM phenocopies, FD, and ATTR-CA variants

		Overall (samples analyzed)	Positives (HCM + HCM phenocopies	HCM	HCM phenocopies	FD	ATTR-CA
Age (years)	N	2068	453	380	73	18	32
	Mean	51.2	46.1	45.1	50.9	51.1	64.6
	SD	18.6	18.7	17.6	23.5	19.4	19.0
	Median	53	46	45	57	51.9	68
	Min	2	2	2	3	4	13
	Max	100	95	83	95	80	95
Sex n (%)	Male	1282 (62.0)	250 (55.2)	204 (53.7)	46 (63.0)	11 (61.1)	21 (65.6)
	Female	783 (37.9)	202 (44.6)	176 (46.3)	26 (35.6)	7 (38.9)	10 (31.3)
	Unknown	3 (0.1)	1 (0.2)	0 (0.0)	1 (1.4)	0 (0.0)	1 (3.1)

*ATTR-CA* Transthyretin cardiac amyloidosis, *FD *Fabry disease, *HCM *Hypertrophic cardiomyopathy, *SD Standard deviation*

**Fig. 1:**
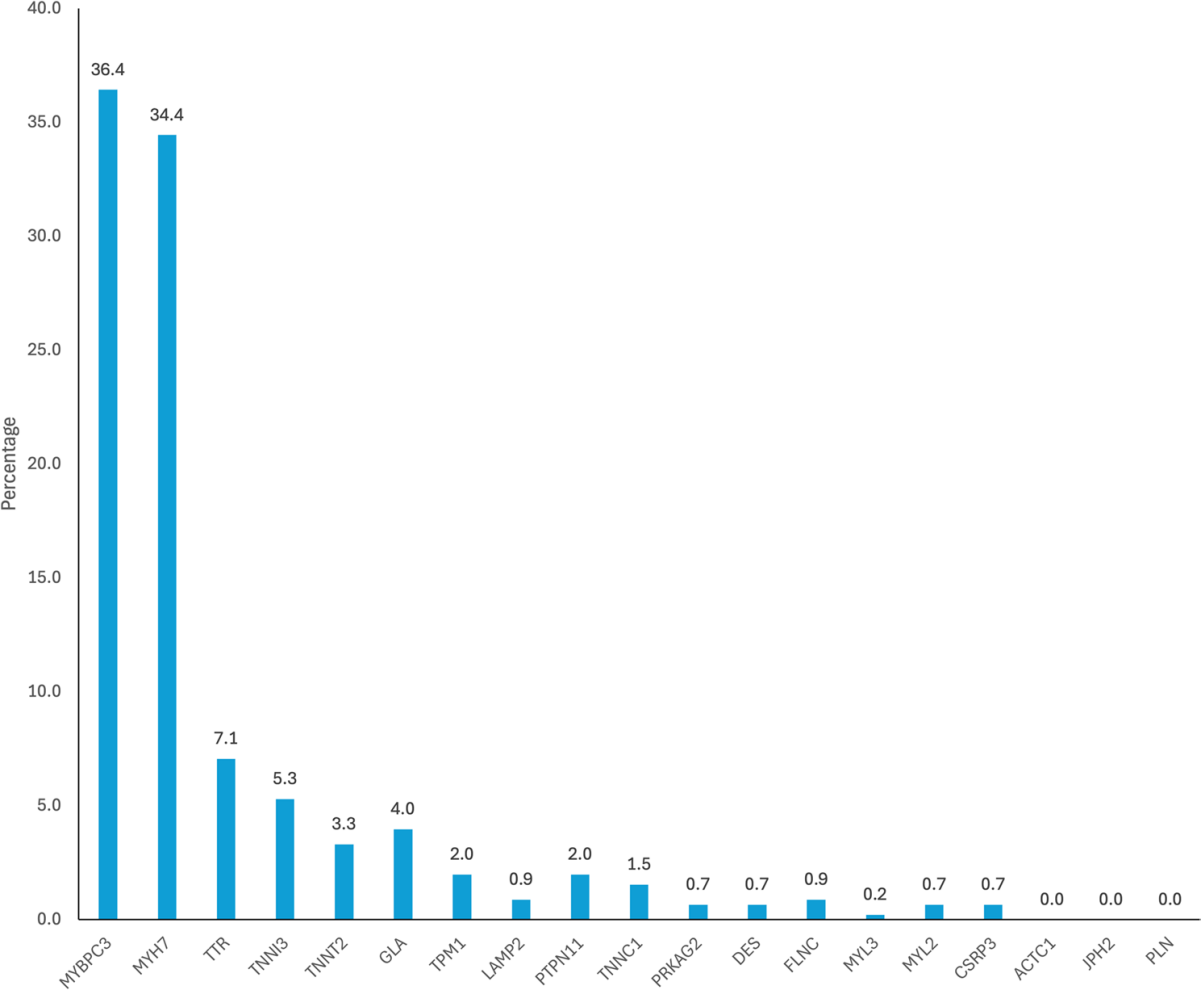
Distribution of patients with confirmed pathogenic/likely pathogenic variants. *ACTC1*, *JPH2*, and *PLN* gene pathogenic variants were not detected in the study population

The genetic analyses conducted for the samples from various countries/regions are presented in Table 3. The largest number of samples came from Colombia (*n* = 651), Brazil (*n* = 501), and Argentina (*n* = 250), collectively accounting for over two-thirds of the total cohort. Brazil (*n* = 150) and Argentina (*n* = 88) contributed the highest number of positive cases, while Brazil also had the highest number of phenocopy cases (*n* = 33). Taiwan (5.3%), Hong Kong (3.9%), and Argentina (2%) showed the highest diagnostic yield for FD, while Brazil (3.8%), Saudi Arabia (3.2%), and Taiwan (2.6%) showed the highest diagnostic yield for ATTR-CA. Overall, 368 healthcare professionals were involved in the study. The Human Gene Mutation Database (HGMD^®^) was used to check the novelty of the identified *GLA* variants. The list of pathogenic/likely pathogenic variants identified is shown in Fig. 1; Table 4. Among the samples analyzed, the most prevalent hereditary HCM was caused by mutations in *MYH7* (34.4%) and *MYBPC3* (36.4%) genes, and the most prevalent phenocopies were caused by mutations in *TTR* (7.1%) and *GLA* (4.0%) genes. The list of patients with pathogenic/likely pathogenic TTR variants is provided in Table 5. The α-Gal A enzyme activity and lyso-Gb3 levels were measured from the DBS samples of patients with pathogenic, likely pathogenic *GLA* variants, and patients with *GLA* VUS, and are presented in Table 6. Most patients (10 of 16 patients with available enzyme data; 62.5%) showed reduced α-Gal A enzyme activity below the diagnostic cut-off of 1.68 µmol/L/h, consistent with Fabry disease diagnosis. Lyso-Gb3 levels were frequently elevated above the reference threshold of 0.8 ng/mL. Among patients with pathogenic/likely pathogenic *GLA* variants, reduced α-Gal A activity was observed in 9 of 10 males with available enzyme data, whereas only 1 of 6 females showed reduced enzyme activity; the remaining females had normal or borderline enzyme levels, several with elevated lyso-Gb3 concentrations, consistent with the known sex-related biochemical variability in Fabry disease. The lists of pathogenic/likely pathogenic variants for HCM and HCM phenocopies across different countries/regions are provided in **additional files 1 and 2**, respectively.

**Table 3:** Frequencies of confirmed molecular diagnosis of HCM, HCM phenocopies, ATTR-CA, and FD

Countries/regions	Total analyzed	No. of patients with pathogenic/likely pathogenic variant (HCM + HCM phenocopies)	No. of patients with HCM variants	No. of patients with HCM phenocopy variants	No. of patients with TTR variants	No. of patients with GLA variants	Overall diagnostic yield (pathogenic/likely pathogenic variants)	Diagnostic yield (ATTR-CA)	Diagnostic yield (FD)
Colombia	651	88	70	18	10	4	13.5%	1.5%	0.6%
Brazil	501	150	117	33	19	3	29.9%	3.8%	0.6%
Argentina	250	88	83	5	0	5	35.2%	0.0%	2.0%
Algeria	93	24	21	3	0	1	25.8%	0.0%	1.1%
Israel	87	8	8	0	0	0	9.2%	0.0%	0.0%
Kazakhstan	90	19	18	1	0	0	21.1%	0.0%	0.0%
Mexico	72	11	8	3	0	1	15.3%	0.0%	1.4%
Hong Kong	51	14	11	3	0	2	27.5%	0.0%	3.9%
Peru	45	9	8	1	1	0	20.0%	2.2%	0.0%
Taiwan	38	11	8	3	1	2	28.9%	2.6%	5.3%
Saudi Arabia	31	2	1	1	1	0	6.5%	3.2%	0.0%
Turkey	34	6	6	0	0	0	17.6%	0.0%	0.0%
UAE	26	5	5	0	0	0	19.2%	0.0%	0.0%
Panama	18	1	1	0	0	0	5.6%	0.0%	0.0%
Chile	18	5	5	0	0	0	27.8%	0.0%	0.0%
Guatemala	16	3	3	0	0	0	18.8%	0.0%	0.0%
Costa Rica	13	3	3	0	0	0	23.1%	0.0%	0.0%
South Africa	11	2	0	2	0	0	18.2%	0.0%	0.0%
El Salvador	10	4	4	0	0	0	40.0%	0.0%	0.0%
Dominican Republic	5	0	0	0	0	0	0.0%	0.0%	0.0%
Ecuador	4	0	0	0	0	0	0.0%	0.0%	0.0%
Kuwait	4	0	0	0	0	0	0.0%	0.0%	0.0%
Total	2068	453*	380	73	32	18	21.9%	1.5%	0.9%

*Among 453 patients, 3 patients had double variants

*ATTR-CA *Transthyretin cardiac amyloidosis, *FD *Fabry disease, *HCM *Hypertrophic cardiomyopathy, *UAE *United Arab Emirates

**Table 4:** Distribution of confirmed pathogenic/likely pathogenic variants

Gene	Number of patients with pathogenic/likely pathogenic variants				Mean age (years)
	Male	Female	Total	%	
*MYBPC3*	91	74	165	36.4	47.88 ± 17.02
*MYH7*	75	81	156	34.4	43.41 ± 17.65
TTR*	21	10	32	7.1	64.56 ± 18.98
*TNNI3*	16	8	24	5.3	47.69 ± 14.15
*TNNT2*	9	6	15	3.3	42.41 ± 20.89
*GLA*	11	7	18	4.0	51.13 ± 19.40
*TPM1*	6	3	9	2.0	34.96 ± 16.69
*LAMP2*	1	3	4	0.9	26.75 ± 5.91
*PTPN11*	5	4	9	2.0	25.00 ± 20.68
*TNNC1*	4	3	7	1.5	32.43 ± 20.55
*PRKAG2*	3	0	3	0.7	39.31 ± 22.49
*DES*	3	0	3	0.7	29.33 ± 1.15
*FLNC*	2	2	4	0.9	47.50 ± 21.02
*MYL3*	0	1	1	0.2	24.00
*MYL2*	2	1	3	0.7	36.33 ± 29.54
*CSRP3*	2	1	3	0.7	54.67 ± 8.74
*ACTC1*	0	0	0	0	0
*JPH2*	0	0	0	0	0
*PLN*	0	0	0	0	0
Total	251	204	456*		

*Sex data not available for one patient (251 males; 204 females; and data on sex of 1 patient not available)

**Table 5:** Patients with pathogenic/likely pathogenic TTR variants

Sl. No.	Age	Sex	DNA	Protein	ACMG classification
1	71	Male	c.424G > A	p.Val142Ile	Likely pathogenic
2	57	Female	c.424G > A	p.Val142Ile	Likely pathogenic
3	68	Male	c.148G > A	p.Val50Met	Pathogenic
4	78	Male	c.424G > A	p.Val142Ile	Likely pathogenic
5	76	Male	c.424G > A	p.Val142Ile	Likely pathogenic
6	88	Male	c.424G > A	p.Val142Ile	Likely pathogenic
7	79	Male	c.424G > A	p.Val142Ile	Likely pathogenic
8	73	Male	c.424G > A	p.Val142Ile	Likely pathogenic
9	67	Male	c.148G > A	p.Val50Me	Pathogenic
10	66	Male	c.209G > A	p.Ser70Asn	Likely pathogenic
11	41	Female	c.424G > A	p.Val142Ile	Likely pathogenic
12*	59	-	c.239 C > T	p.Thr80Ile	Pathogenic
13	74	Female	c.209G > A	p.Ser70Asn	Likely pathogenic
14	95	Female	c.424G > A	p.Val142Ile	Likely pathogenic
15	73	Male	c.424G > A	p.Val142Ile	Likely pathogenic
16	38	Male	c.148G > A	p.Val50Met	Pathogenic
17	74	Male	c.424G > A	p.Val142Ile	Likely pathogenic
18	33	Male	c.424G > A	p.Val142Ile	Likely pathogenic
19	68	Male	c.148G > A	p.Val50Met	Pathogenic
20	74	Male	c.148G > A	p.Val50Met	Pathogenic
21	86	Female	c.424G > A	p.Val142Ile	Likely pathogenic
22	63	Male	c.424G > A	p.Val142Ile	Likely pathogenic
23	64	Female	c.424G > A	p.Val142Ile	Likely pathogenic
24	68	Female	c.424G > A	p.Val142Ile	Likely pathogenic
25	63	Female	c.424G > A	p.Val142Ile	Likely pathogenic
26	68	Male	c.258 A > T	p.Glu86Asp	Pathogenic
27	79	Male	c.424G > A	p.Val142Ile	Likely pathogenic
28	72	Male	c.424G > A	p.Val142Ile	Likely pathogenic
29	22	Male	c.424G > A	p.Val142Ile	Likely pathogenic
30	13	Male	c.424G > A	p.Val142Ile	Likely pathogenic
31	35	Female	c.424G > A	p.Val142Ile	Likely pathogenic
32	80	Female	c.424G > A	p.Val142Ile	Likely pathogenic

*Sex data was not available for one patient

*ACMG *American College of Medical Genetics and Genomics,* DNA *Deoxyribonucleic Acid

**Table 6:** α-Gal A activity and lyso-Gb3 levels in patients with pathogenic/likely pathogenic variants

Sl. no.	Age (years)	Sex	Enzyme activity level (cut-off levels: 1.68–13.63 µmol/L/h)	Lyso-Gb3 (cut-off levels: 0.8 ng/mL)	Fabry variants		
					DNA	Protein	ACMG classification
1	4	Male	-	-	c.520T > G	p.Cys174Gly	Likely pathogenic
2	61	Male	0.52	3.9	c.644 A > G	p.Asn215Ser	Pathogenic
3	36	Male	1.59	1.1	c.525 C > A	p.Asp175Glu	Likely pathogenic
4	68	Male	0.88	< 0.5	c.870G > C	p.Met290Ile	Pathogenic
5	53	Male	0.26	2.6	c.520T > G	p.Cys174Gly	Likely pathogenic
6	52	Male	0.17	5.3	c.1066 C > T	p.Arg356Trp	Pathogenic
7	62	Male	10.28	2.1	c.1088G > A	p.Arg363His	Pathogenic
8	44	Male	0.26	24	c.50_54dup	p.Leu19Alafs*104	Likely pathogenic
9	67	Male	0.63	2.9	c.640-801G > A	Splicing	Pathogenic
10	52	Male	0.13	6.7	c.1066 C > T	p.Arg356Trp	Pathogenic
11	43	Male	0.18	8.4	c.1066 C > T	p.Arg356Trp	Pathogenic
12	71	Female	-	-	c.41T > C	p.Leu14Pro	Pathogenic
13	35	Female	2.44	0.7	c.870G > C	p.Met290Ile	Pathogenic
14	22	Female	0.75	2	c.644 A > G	p.Asn215Ser	Pathogenic
15	46	Female	1.86	2	c.419 A > C	p.Lys140Thr	Likely pathogenic
16	47	Female	2.97	1.5	c.640-801G > A	Splicing	Pathogenic
17	78	Female	12.09	2.2	c.520T > G	p.Cys174Gly	Likely pathogenic
18	80	Female	2.24	1.5	c.640-801G > A	Splicing	Pathogenic

α-Gal A enzyme activity and lyso-Gb3 levels of patient 1 and patient 12 were not estimated due to the poor quality of DBS samples

*ACMG *American College of Medical Genetics and Genomics, *DBS *Dried blood spot, *DNA *Deoxyribonucleic Acid, *GLA *Gamma-linolenic acid, *Lyso-Gb3 *Globotriaosylsphingosine

Of the 2068 analyzed samples, 3 patients had pathogenic variants in two different genes (2 females, 1 male), Table 7. Double variants were seen in 1 patient each from Hong Kong, Brazil, and Kazakhstan.

**Table 7:** Double mutations in patients with unexplained LVWT

Number of samples with double mutations (*n* = 3)	Country/region	Age (years)	Sex	Gene 1	Class 1	Gene 2	Class 2
1	Hong Kong	46	Male	*TNNI3* (c.485G > A)	Pathogenic	*MYBPC3* (c.1224-19G > A)	Pathogenic
1	Kazakhstan	21	Female	*MYBPC3* (c.693dupT)	Pathogenic	*MYH7* (c.1370T > C)	Pathogenic
1	Brazil	34	Female	*MYBPC3* (c.1484G > A)	Pathogenic	*MYH7* (c.1988G > A)	Pathogenic

*LVWT* Left ventricular wall thickness

## Discussion

HCM is a genetically heterogeneous autosomal-dominant disorder [10, 21]. It poses significant diagnostic challenges due to its clinical variability and overlap with phenocopies such as FD and ATTR-CA [15, 22]. The possibility of having alternate inheritance modes, additional clinical features, variable treatment options, and a different prognosis underscores the need for accurate diagnosis of diseases that mimic HCM [23]. This study investigated the detection rate of variants related to HCM or its phenocopies, utilizing a 19-gene NGS panel. This was applied to a global cohort of patients with unexplained increased LVWT from 22 countries/regions. The region-wise prevalence of variants associated with hypertrophic phenotypes and their phenocopies was also evaluated. Compared with the previous cardio NGS pilot study (*n* = 535), the present study includes a substantially larger cohort (*n* = 2068) from a broader geographic distribution, increasing the precision of prevalence estimates and enabling more robust evaluation of phenocopies across diverse populations.

Our study, involving 2068 samples, revealed a detection rate of 21.9% (453/2068) for pathogenic/likely pathogenic variants associated with HCM and its phenocopies. This includes the identification of pathogenic/likely pathogenic *TTR* variants in 32 patients (1.5%) and *GLA* variants in 18 patients (0.9%). The diagnostic yield of NGS screening observed in this study is relatively lower than that from previously published studies, which reported a range between 21% and 79% for HCM and 5%–10% for HCM phenocopies [18]. Importantly, genetic diagnostic yield in HCM is known to vary substantially depending on the clinical context. In younger patients with a familial background or a pronounced phenotype, genetic testing yields reach approximately 60%–70%, whereas in older individuals presenting with sporadic or less pronounced phenotypes, the diagnostic yield declines to approximately 15%–30% [24–27]. In this study, broad inclusion criteria were applied based on unexplained increased LVWT (≥ 12 mm), thereby capturing a heterogeneous, phenotype-driven population rather than a highly selected referral cohort with clinically definite HCM according to guideline criteria. This broader sampling strategy explains the lower overall genetic yield observed when compared with the yields reported in cohorts enriched for early-onset, familial, or clinically unequivocal HCM. While this broader inclusion strategy may have contributed to the modestly lower overall genetic yield (21.9% vs. 24.7% in our earlier cohort), it allowed enhanced identification of clinically actionable phenocopies. Notably, the detection rate of TTR-related amyloidosis increased from 1.3% to 1.5%, and the yield of Fabry disease increased from 0.4% to 0.9%. Because these conditions often present with milder or early-stage hypertrophy, expanding the LVWT threshold enabled identification of additional treatable cases, reinforcing the clinical relevance of this broader inclusion strategy [28]. The findings of this study are congruent with the results of the cardio NGS pilot study, which concluded that the most prevalent genetic variants for HCM were *MYH7* and *MYBPC3*, and the most prevalent variant for the HCM phenocopy was *TTR* [18]. Expanding the panel with *CSRP3* led to identification of three pathogenic variants, however, no pathogenic/likely pathogenic variants were detected in *JPH2*. The modestly lower proportion of detected sarcomeric HCM-associated variants in the current study than in the previous one may reflect the inclusion of patients with borderline or mild hypertrophy compared to our previous study, where the likelihood of classic sarcomeric mutations is lower. These findings suggest that while genetic testing remains informative, the diagnostic yield for HCM variants may decrease when extending testing to patients with less overt wall thickening. On the other hand, the broader inclusion criteria compared to the previous study seemed to slightly enhance the identification of phenocopies. This likely reflects the subclinical or atypical hypertrophy seen in phenocopy conditions, where wall thickness alone is insufficient for differentiation. In such scenarios, NGS panels that include genes like *GLA* and *TTR* become particularly valuable for early diagnosis and timely intervention. In addition, HCM caused by double sarcomere gene variants was identified in 3 patients in the current study. Two of these patients had pathogenic variants in both the *MYH7* and *MYBPC3* genes, while the third patient had pathogenic variants in the *TNNI3* and *MYBPC3* genes. Two separate mutations within the same or different sarcomere genes have been reported in 3%–6% of consecutively screened cohorts [14]. Earlier onset and more severe clinical profiles of HCM have been associated with double mutations compared to a single mutation. The high prevalence of *MYBPC3* (165 variants) and *MYH7* (156 variants) variants highlights these genes as major contributors to the HCM phenotype, as reported previously [29]. This indicates that the variants described here may be the underlying cause for a substantial number of HCM cases in the countries/regions included in this study.

A total of 20 novel variants were detected. Novel variants have been marked in additional files 1 and 2. Among the HCM-related genes, a total of 16 novel variants were identified (13 in *MYBPC3*, 2 in *MYH7*, 1 in *TNNI3*). *MYBPC3* variant c.1897G > T, variant c.1449_1455delAGTCAAA and variant c.729_735delCAAGGAC were from Argentina, variant c.1184_1185delAA, variant c.1587dupT and variant c.1358_1359delCT were from Colombia, variant c.1535_1538del TGAT and variant c.496delG were from Taiwan, variant c.1486G > T was from Israel, variant c.693dupT and c.350dupC were from Kazakhstan, variant c.3309delG was from Peru, and variant c.121delC was from Hong Kong. *MYH7* variant c.2092G > T was from Hong Kong, variant c.2713T > C was from Colombia, and *TNNI3* variant c.336 C > A was from Algeria. Among HCM phenocopies, 2 novel variants were identified (1 in *LAMP2*, 1 in *GLA*). *LAMP2* variant c.869dupA was from Colombia, *GLA* variant c.50_54dup GCTTC was from Argentina. In addition, we identified 2 pathogenic variants in *FLNC*: variant c.3562delC from Hong Kong, and variant c.2813delG from Brazil. The diversity and clinical implications of these novel variants likely reflect the underrepresentation of these populations in previous genetic studies.

There are few studies on targeted sequencing of broad cardiomyopathy gene panels in large populations of patients with HCM. In addition, variability in patient cohorts, methodologies, and interpretations of results makes cross-study comparisons challenging [30]. Our study, however, reports the frequency of pathogenic variants among a large pool of unrelated HCM patients across 22 countries/regions who were screened for variations in 19 genes. By providing data from a geographically diverse cohort, this study contributes to a better understanding of the distribution of sarcomeric and phenocopy-related variants. This is in line with the 2025 clinical consensus statement from the ESC council on cardiovascular genomics, which recommends the use of phenotype-directed multigene panels as the first-line diagnostic strategy for cardiomyopathies [31].

Recent insights from the Laboratory of Molecular Medicine (LMM) suggest that using expanded gene panels beyond the primary 11 sarcomeric and metabolic cardiomyopathy genes provides only a slight improvement in test yield and sensitivity. When combining clinical genetics findings from LMM and the Oxford Molecular Genetics Laboratory, it is evident that over 99% of variants identified as pathogenic or likely pathogenic based on clinical-grade criteria for HCM testing are linked to the eight core sarcomeric genes: *MYH7*, *MYBPC3*, *TNNT2*, *TPM1*, *MYL2*, *MYL3*, *TNNI3*, and *ACTC1* [32]. Similarly, it has been indicated in another study that expanded gene panels do not result in a substantial increase in HCM sensitivity testing beyond gene panels with eight core sarcomeric genes, *GLA*, *PRKAG2*, and *LAMP2* [2].

The presence of *TTR* variants in 32 patients underscores the importance of considering cardiac amyloidosis in differential diagnoses, while the identification of *GLA* variants in 18 patients highlights the need to also consider FD. The frequency of variants of the *GLA* gene in patients with HCM can be as high as 6.7% [33]. A meta-analysis encompassing over 10,000 patients with left ventricular hypertrophy (LVH)/HCM found that FD has a prevalence rate of 1.2% [34]. Additionally, a meta-analysis that compiled results from two studies focusing on older HCM patient cohorts indicates that the prevalence of cardiac amyloidosis ranges between 5% and 9% [35]. However, it is crucial to consider that these prevalence rates might be influenced by referral bias, given that the data predominantly originated from amyloidosis referral centers [9]. The findings from this study emphasize the importance of the NGS panel test for identifying disorders with overlapping clinical manifestations and its capacity to detect phenocopies that otherwise would be missed by common imaging studies. Overall, promoting genetic testing for HCM and its phenocopies, including FD and ATTR-CA, among a representative global population is an urgent need, along with creating awareness about FD, ATTR-CA, and its overlapping HCM phenotypes. As an addition to our previous study, quantitative reporting of α-Gal A enzyme activity and lyso-Gb3 levels was included here. The cohort of 18 individuals carrying *GLA* gene variants demonstrates the substantial biochemical and genetic heterogeneity characteristic of FD. Most patients with pathogenic or likely pathogenic variants showed markedly reduced α-Gal A enzyme activity below the reference cut-off and elevated lyso-Gb3 concentrations, consistent with Fabry disease diagnosis. However, some patients, especially females, demonstrated normal enzyme activity despite elevated lyso-Gb3 levels, indicating variability. This combined approach of DBS-based enzyme activity measurement and lyso-Gb3 quantification with NGS can improve diagnostic accuracy and help identify patients with atypical or later-onset FD who might otherwise be overlooked [36, 37].

The mean age of patients with pathogenic/likely pathogenic variants was 46.1 ± 18.7 years; 18 *GLA* variants were identified with a mean age of 51.1 ± 19.4 years, and 32 patients with *TTR* variants were identified with a mean age of 64.6 ± 19.0 years. In the previous cardio NGS pilot study, the mean ages of patients with HCM or HCM phenocopies, FD, and ATTR-CA were 42.8 ± 17.9, 69.0 ± 1.4, and 54.6 ± 17.0 years, respectively [18]. The mean age of patients with HCM and its phenocopies suggests that there is still a delay in the diagnosis of these patients until the disease has progressed significantly, highlighting the need for increased clinical awareness and early screening, especially for patients presenting with unexplained increased LVWT. The use of NGS panels in this context proves invaluable, as conventional imaging techniques might not distinguish between HCM and its phenocopies [38]. The use of NGS panels is crucial in distinguishing HCM from its phenocopies, such as FD and ATTR-CA, because these entities have specific cardiac and extracardiac clinical features, complications, and therapies. For FD, measuring alpha-Gal A activity and lyso-Gb3 levels followed by *GLA* gene sequencing provides a robust diagnostic approach and should be used in clinical practice if disease is suspected or when NGS panels are not available [36, 37]. For ATTR-CA, *TTR* gene sequencing identifies hereditary ATTR and supports etiological classification and family screening; however, definitive diagnosis requires confirmatory imaging with bone tracer scintigraphy or endomyocardial biopsy [39].

ATTR-CA and FD are progressive disorders with serious complications, and thereby, accurate diagnosis is crucial for accurate management and timely initiation of specific treatments in these groups of patients. Moreover, with the development of disease-modifying agents that have been reported to show better outcomes in early-stage ATTR-CA, it is pertinent to screen patients with increased LVWT of unknown origin for cardiac amyloidosis [39]. Similarly, early treatment for FD with ERT has been reported to enhance clinical outcomes for patients when started early [40, 41].

When the data were analyzed region-wise, significant variations in the prevalence of genetic variants were observed. For instance, Brazil exhibited a high diagnostic yield of 29.9%. Countries such as Kuwait and the Dominican Republic showed no detected variants; however, this may be due to the small cohort size. Regions with the highest diagnostic yield for FD included Taiwan, Hong Kong, and Argentina, while the diagnostic yield for ATTR-CA was highest in Brazil, Saudi Arabia, and Taiwan. These differences could reflect differences in genetic backgrounds, healthcare accessibility, and regional epidemiology. Variability in the HCM genetic background across different populations has been previously suggested [30]. The identification of regional prevalence patterns is also crucial for developing tailored screening programs and healthcare policies that address specific population needs. Selecting patients appropriately can help enhance the genetic testing yield and improve the cost-effectiveness of diagnostic testing [22].

This study has some limitations that must be considered when interpreting the results. The diagnostic yield of this study was lower than in most HCM cohorts. The 12-mm wall thickness inclusion threshold could explain a higher proportion of hypertensive heart disease and other cases of secondary increased LVWT instead of genetically determined sarcomeric cardiomyopathy. Further, the inclusion criterion of LVWT ≥ 12 mm could not be strictly verified for all patients, which may have affected the consistency of sample selection. No numeric wall thickness data were available to corroborate these assumptions or to stratify patients by specific LVWT intervals to determine whether individuals with a wall thickness of 12 mm carried pathogenic or likely pathogenic variants. The absence of detailed phenotypic data for the samples limits the ability to establish clear genotype–phenotype correlations. Although pediatric patients were included in the study, they represented a small proportion of the cohort. Due to the limited number of pediatric cases and the absence of uniform, age-stratified phenotypic data across participating centers, dedicated pediatric subgroup analyses were not feasible. Consequently, age-specific differences in disease severity or phenotype could not be systematically evaluated. Although the study includes analysis of α-Gal A enzyme activity and lyso-Gb3 levels in identified FD patients, more comprehensive phenotypic characterization would enhance the utility of the genetic findings. Secondly, a 19-gene panel was used in this study, which did not cover all HCM-associated genes, potentially missing some disease-causing variants such as *FHOD3*, *TRIM63*, and *FHL1*, which have been more recently identified, and the understanding of their role in HCM pathogenesis is still evolving.

## Conclusion

This global multicentric study confirms the findings of the previous cardio NGS pilot study, demonstrating the critical importance of implementing the NGS strategies to enhance the diagnosis of patients with HCM, and phenocopies of HCM, including ATTR-CA and FD. By encompassing a diverse patient population from 22 countries/regions, this study underscores the effectiveness of NGS in genetic testing on a larger scale. The findings highlight the necessity of incorporating *GLA* and *TTR* in all cardio NGS panels to facilitate the early diagnosis of FD and ATTR-CA, which are conditions that often present with overlapping clinical features and require different and specific treatment approaches that may provide better patient outcomes. Given the high diagnostic yield and the clinical benefits of early detection, we propose that NGS be employed as a front-line diagnostic tool for individuals with unexplained increased LVWT. In regions where NGS panels may not be readily available, alternative diagnostic measures should be taken to exclude ATTR-CA or FD in patients with unexplained LVWT to enable earlier treatment and improve patient outcomes. 

## Supplementary Information

Supplementary Material 1.

Supplementary Material 2.

## Data Availability

All data that do not infringe upon the rights and privacy of the individuals who participated in the study are available upon reasonable request, in accordance with Sanofi’s data-sharing guidelines. Requests can be directed to the corresponding author at [huseyin.onay@multigen.com.tr](mail to: huseyin.onay@multigen.com.tr).

## Abbreviations

α-Gal A: α-galactosidase A
ATTR-CA: Transthyretin cardiac amyloidosis
DBS: Dried blood spot
ERT: Enzyme replacement therapy
FD: Fabry disease
FIA-MS/MS: Flow injection analysis tandem mass spectrometry
HCM: Hypertrophic cardiomyopathy
LC-MS/MS: Liquid chromatography-tandem mass spectrometry
LMM: Laboratory of Molecular Medicine
LVWT: Left ventricular wall thickness
LVH: Left ventricular hypertrophy
lyso-Gb3: Globotriaosylsphingosine
MRM: Multiple reaction monitoring
NGS: Next-generation sequencing
VUS: Variants of unknown significance
