# Supplementary material for: Uncovering the gene variants in a global cohort of patients with unexplained increased left ventricular wall thickness using next-generation sequencing
Source: BMC Cardiovasc Disord. 2026 Apr 17;26:463. doi: 10.1186/s12872-026-05834-5 (PMC13231718; doi:10.1186/s12872-026-05834-5)
Supplement: Supplementary file 1 — Supplementary Material 1. [file 12872_2026_5834_MOESM1_ESM.docx]

**Supplementary Table S1:** Classifications of pathogenicity and nucleotide/amino acid changes for all hypertrophic cardiomyopathy positive variants

| **Sl. No.** | **Gene** | | **Number of patients** | **Nucleotide change** | **Reference SNP (rs) No** | **ACMG classification** | **Amino acid change** | **Novel HGMD/ClinVar**  **(+)=known (-)novel** | **Countries** |
| --- | --- | --- | --- | --- | --- | --- | --- | --- | --- |
| 1 | *MYBPC3* | | 19 | c.1484G>A | rs200411226 | Pathogenic | Arg495Gln | +/+ (P) | Brazil (19) |
| 2 | *MYBPC3* | | 8 | c.1928-2A>G | [rs397515937](http://www.ncbi.nlm.nih.gov/snp/rs397515937) | Pathogenic | splicing | +/+ (P) | Argentina (8) |
| 3 | *MYBPC3* | | 8 | c.772G>A | [rs397516074](http://www.ncbi.nlm.nih.gov/snp/rs397516074) | Likely Pathogenic | Glu258Lys | +/+ (P) | Argentina (3), Brazil (2), Colombia (3) |
| 4 | *MYBPC3* | | 6 | c.2308G>A | [rs36211723](http://www.ncbi.nlm.nih.gov/snp/rs36211723) | Pathogenic | Asp770Asn | +/+ (P-LP) | Brazil (2), El Salvador (4) |
| 5 | *MYBPC3* | | 8 | c.2670G>A | [rs397515982](http://www.ncbi.nlm.nih.gov/snp/rs397515982) | Pathogenic | Trp890* | +/+ (P-LP) | Colombia (8) |
| 6 | *MYBPC3* | | 5 | c.1227-13G>A | [rs397515893](http://www.ncbi.nlm.nih.gov/snp/rs397515893) | Likely Pathogenic | splicing | +/+ (P-LP) | Algeria (2), Argentina (2), Colombia (1) |
| 7 | *MYBPC3* | | 4 | c.1513_1515delAAG | [rs727504287](http://www.ncbi.nlm.nih.gov/snp/rs727504287) | Pathogenic | Lys505del | +/+ (P-LP) | Argentina (1), Brazil (1), Colombia (1), Mexico (1) |
| 8 | *MYBPC3* | | 4 | c.3190+1G>A | [rs111683277](http://www.ncbi.nlm.nih.gov/snp/rs111683277) | Pathogenic | splicing | +/+ (P) | Argentina (2), Israel (2) |
| 9 | *MYBPC3* | | 3 | c.237C>G | [rs730880698](http://www.ncbi.nlm.nih.gov/snp/rs730880698) | Pathogenic | Tyr79* | +/+ (P) | Argentina (3) |
| 10 | *MYBPC3* | | 3 | c.2670dupG | [rs863225104](http://www.ncbi.nlm.nih.gov/snp/rs863225104) | Pathogenic | Arg891Alafs*160 | +/+ (LP) | Argentina (1), Brazil (2) |
| 11 | *MYBPC3* | | 3 | c.1624G>C | [rs121909374](http://www.ncbi.nlm.nih.gov/snp/rs121909374) | Pathogenic | Glu542Gln | +/+ (P) | Argentina (2), Colombia (1) |
| 12 | *MYBPC3* | | 3 | c.913_914delTT | [rs397516080](http://www.ncbi.nlm.nih.gov/snp/rs397516080) | Pathogenic | Phe305Profs*27 | +/+ (P) | Brazil (3) |
| 13 | *MYBPC3* | | 3 | c.1505G>A | [rs397515907](http://www.ncbi.nlm.nih.gov/snp/rs397515907) | Pathogenic | Arg502Gln | +/+ (P) | Algeria (1), Brazil (2) |
| 14 | *MYBPC3* | | 3 | c.1483C>T | [rs397515905](http://www.ncbi.nlm.nih.gov/snp/rs397515905) | Pathogenic | Arg495Trp | +/+ (P/LP) | Colombia (3) |
| 15 | *MYBPC3* | | 3 | c.3746dupG | N/A | Likely Pathogenic | Ile1250Hisfs*16 | +/+ (P/P) | Brazil (1), Colombia (2) |
| 16 | *MYBPC3* | | 2 | c.2905+1G>A | [rs397515991](http://www.ncbi.nlm.nih.gov/snp/rs397515991) | Pathogenic | splicing | +/+ (P) | Argentina (2) |
| 17 | *MYBPC3* | | 2 | c.1897G>T | N/A | Likely Pathogenic | Glu633* | -/-** | Argentina (2) |
| 18 | *MYBPC3* | | 2 | c.1790G>A | [rs727503195](http://www.ncbi.nlm.nih.gov/snp/rs727503195) | Pathogenic | Arg597Gln | +/+ (P/LP) | Costa Rica (2) |
| 19 | *MYBPC3* | | 2 | c.1184_1185delAA | N/A | Likely Pathogenic | Lys395Metfs*18 | -/-** | Colombia (2) |
| 20 | *MYBPC3* | | 2 | c.1119C>G | N/A | Pathogenic | Tyr373* | -/+ (P) | Colombia (2) |
| 21 | *MYBPC3* | | 2 | c.1449_1455delAGTCAAA | N/A | Likely Pathogenic | Gln483Hisfs*3 | -/-** | Argentina (2) |
| 22 | *MYBPC3* | | 2 | c.1535_1538del TGAT | N/A | Likely Pathogenic | Leu512Profs*42 | -/-** | Taiwan (2) |
| 23 | *MYBPC3* | | 2 | c.3662delT | [rs863225107](http://www.ncbi.nlm.nih.gov/snp/rs863225107) | Pathogenic | Leu1221Argfs*16 | +/+ (P) | Brazil (2) |
| 24 | *MYBPC3* | | 2 | c.1999_2000delCTinsG | [rs727503192](http://www.ncbi.nlm.nih.gov/snp/rs727503192) | Pathogenic | Leu667Aspfs*15 | +/+ (P) | Costa Rica (1), Guatemala (1) |
| 25 | *MYBPC3* | | 3 | c.1800delA | [rs397515926](http://www.ncbi.nlm.nih.gov/snp/rs397515926) | Pathogenic | Lys600Asnfs*2 | +/+ (P) | Brazil (1), Mexico (2) |
| 26 | *MYBPC3* | | 2 | c.362delC | [rs397516032](http://www.ncbi.nlm.nih.gov/snp/rs397516032) | Pathogenic | Pro121Argfs*38 | +/+ (P) | Argentina (2) |
| 27 | *MYBPC3* | | 2 | c.1504C>T | [rs375882485](http://www.ncbi.nlm.nih.gov/snp/rs375882485) | Pathogenic | Arg502Trp | +/+ (P) | Brazil (2) |
| 28 | *MYBPC3* | | 2 | c.2149-1G>A | [rs727504334](http://www.ncbi.nlm.nih.gov/snp/rs727504334) | Pathogenic | splicing | + (VUS)/+ (P) | Argentina (2) |
| 29 | *MYBPC3* | | 1 | c.2893C>T | [rs730880578](http://www.ncbi.nlm.nih.gov/snp/rs730880578) | Pathogenic | Gln965Ter | +/+ (P) | Kazakhstan (1) |
| 30 | *MYBPC3* | | 1 | c.2992C>T | [rs11570112](http://www.ncbi.nlm.nih.gov/snp/rs11570112) | Pathogenic | Gln998* | +/+ (P) | Colombia (1) |
| 31 | *MYBPC3* | | 1 | c.2670G>A | [rs397515982](http://www.ncbi.nlm.nih.gov/snp/rs397515982) | Pathogenic | Trp890* | +/+ (P) | Colombia (1) |
| 32 | *MYBPC3* | | 1 | c.3697C>T | [rs397516037](http://www.ncbi.nlm.nih.gov/snp/rs397516037) | Pathogenic | Gln1233* | +/+ (P) | Israel (1) |
| 33 | *MYBPC3* | | 1 | c.2450G>A | [rs397515964](http://www.ncbi.nlm.nih.gov/snp/rs397515964) | Pathogenic | Arg817Gln | +(VUS)/+(VUS/P) | Algeria (1) |
| 34 | *MYBPC3* | | 1 | c.2710delT | N/A | Likely Pathogenic | Tyr904Thrfs*20 | +/- | Argentina (1) |
| 35 | *MYBPC3* | | 1 | c.2097delA | [rs869025460](http://www.ncbi.nlm.nih.gov/snp/rs869025460) | Pathogenic | Asp700Metfs*54 | -/+ (LP) | Argentina (1) |
| 36 | *MYBPC3* | | 1 | c.1522C>T | [rs730880544](http://www.ncbi.nlm.nih.gov/snp/rs730880544) | Pathogenic | Gln508* | +/+ (P/LP) | Taiwan (1) |
| 37 | *MYBPC3* | | 2 | c.1587dupT | N/A | Likely Pathogenic | Ser530* | -/-** | Colombia (2) |
| 38 | *MYBPC3* | | 1 | c.1358_1359delCT | N/A | Likely Pathogenic | Pro453Argfs*21 | -/-** | Colombia (1) |
| 39 | *MYBPC3* | | 1 | c.226C>T | [rs1383067193](http://www.ncbi.nlm.nih.gov/snp/rs1383067193) | Pathogenic | Gln76* | +/+ (P) | Argentina (1) |
| 40 | *MYBPC3* | | 1 | c.1303C>T | [rs1432810664](http://www.ncbi.nlm.nih.gov/snp/rs1432810664) | Pathogenic | Gln435* | +/+ (P) | Algeria (1) |
| 41 | *MYBPC3* | | 1 | c.2550delC | [rs863225105](http://www.ncbi.nlm.nih.gov/snp/rs863225105) | Pathogenic | Asn850Lysfs*29 | -/+ (P/LP) | Brazil (1) |
| 42 | *MYBPC3* | | 1 | c.1224-19G>A | [rs587776699](http://www.ncbi.nlm.nih.gov/snp/rs587776699) | Likely Pathogenic | splicing | +/+ (P/LP/VUS) | Hong Kong (1) |
| 43 | *MYBPC3* | | 1 | c.1227-13G>A | [rs397515893](http://www.ncbi.nlm.nih.gov/snp/rs397515893) | Likely Pathogenic | (?) | +/+ (P) | Algeria (1) |
| 44 | *MYBPC3* | | 1 | c.1624+4A>T | [rs397515916](http://www.ncbi.nlm.nih.gov/snp/rs397515916) | Likely Pathogenic | splicing | +/+ (P) | Argentina (1) |
| 45 | *MYBPC3* | | 1 | c.208delG | N/A | Likely Pathogenic | Glu70Lysfs*26 | +/- | Colombia (1) |
| 46 | *MYBPC3* | | 1 | c.2234A>G | [rs727503190](http://www.ncbi.nlm.nih.gov/snp/rs727503190) | Likely Pathogenic | Asp745Gly | +/+ (P/VUS) | Brazil (1) |
| 47 | *MYBPC3* | | 1 | c.3330+5G>C | [rs373746463](http://www.ncbi.nlm.nih.gov/snp/rs373746463) | Likely Pathogenic | ? | +/+ (P) | Colombia (1) |
| 48 | *MYBPC3* | | 1 | c.729_735delCAAGGAC | N/A | Likely Pathogenic | Lys244Asnfs*54 | -/-** | Argentina (1) |
| 49 | *MYBPC3* | | 1 | c.3767_3769del CCA | [rs397516040](http://www.ncbi.nlm.nih.gov/snp/rs397516040) | Likely Pathogenic | Thr1256del | +/+ (LP) | Algeria (1) |
| 50 | *MYBPC3* | | 1 | c.133G>T | N/A | Likely Pathogenic | Gly45* | -/+(LP) | Brazil (1) |
| 51 | *MYBPC3* | | 1 | c.3811C>T | [rs397516042](http://www.ncbi.nlm.nih.gov/snp/rs397516042) | Pathogenic | Arg1271* | +/+ (P) | Colombia (1) |
| 52 | *MYBPC3* | | 1 | c.3617G>A | [rs1057517769](http://www.ncbi.nlm.nih.gov/snp/rs1057517769) | Likely Pathogenic | Gly1206Asp | +/+ (VUS-P) | Brazil (1) |
| 53 | *MYBPC3* | | 1 | c.1486G>T | N/A | Likely Pathogenic | Glu496* | -/-** | Israel (1) |
| 54 | *MYBPC3* | | 1 | c.1377delC | [rs786204339](http://www.ncbi.nlm.nih.gov/snp/rs786204339) | Pathogenic | Leu460Trpfs*6 | +/+ (P) | Taiwan (1) |
| 55 | *MYBPC3* | | 1 | c.3192dupC | [rs397516007](http://www.ncbi.nlm.nih.gov/snp/rs397516007) | Pathogenic | Lys1065Glnfs*12 | +/+ (P) | Argentina (1) |
| 56 | *MYBPC3* | | 1 | c.693dupT | N/A | Likely Pathogenic | Ala232Cysfs*9 | -/-** | Kazakhstan (1) |
| 57 | *MYBPC3* | | 1 | c.1409G>A | [rs776734314](http://www.ncbi.nlm.nih.gov/snp/rs776734314) | Likely Pathogenic | Arg470Gln | +/+ (VUS-LP) | Kazakhstan (1) |
| 58 | *MYBPC3* | | 1 | c.2645_2646del | N/A | Pathogenic | Ser882Ter | -/+ (P) | Argentina (1) |
| 59 | *MYBPC3* | | 1 | c.3641G>A | [rs730880597](http://www.ncbi.nlm.nih.gov/snp/rs730880597) | Pathogenic | Trp1214* | +/+ (P) | Brazil (1) |
| 60 | *MYBPC3* | | 1 | c.1352-2_1353dup AGAG | N/A | Likely Pathogenic | Glu451ArgfsTer17 | -/+ (VUS) | Chile (1) |
| 61 | *MYBPC3* | | 1 | c.496delG | N/A | Likely Pathogenic | Val166* | -/-** | Taiwan (1) |
| 62 | *MYBPC3* | | 1 | c.1090G>A | [rs794727046](http://www.ncbi.nlm.nih.gov/snp/rs794727046) | Pathogenic | Ala364Thr | +/+ (P-LP) | Argentina (1) |
| 63 | *MYBPC3* | | 1 | c.3732C>A | [rs730880600](http://www.ncbi.nlm.nih.gov/snp/rs730880600) | Pathogenic | Cys1244* | +/+ (P) | Argentina (1) |
| 64 | *MYBPC3* | | 1 | c.3182_3190+4del AGGTTGTTGGTGC | [rs730880718](http://www.ncbi.nlm.nih.gov/snp/rs730880718) | Pathogenic | ? | +/+ (P) | Argentina (1) |
| 65 | *MYBPC3* | | 1 | c.3309delG | N/A | Likely Pathogenic | Ala1105Profs*84 | -/-** | Peru (1) |
| 66 | *MYBPC3* | | 1 | c.1591G>A | [rs397515912](http://www.ncbi.nlm.nih.gov/snp/rs397515912) | Pathogenic | Gly531Arg | +/+ (LP) | Peru (1) |
| 67 | *MYBPC3* | | 1 | c.2429G>A | [rs375675796](http://www.ncbi.nlm.nih.gov/snp/rs375675796) | Pathogenic | Arg810His | +/+ (LP-VUS) | Brazil (1) |
| 68 | *MYBPC3* | | 1 | c.121delC | N/A | Likely Pathogenic | Arg41Alafs*26 | -/-** | Hong Kong (1) |
| 69 | *MYBPC3* | | 1 | c.901A>T | [rs730880629](http://www.ncbi.nlm.nih.gov/snp/rs730880629) | Pathogenic | Lys301* | +/+ (P) | Guatemala (1) |
| 70 | *MYBPC3* | | 1 | c.906-36G>A | [rs864622197](http://www.ncbi.nlm.nih.gov/snp/rs864622197) | Pathogenic | splicing | +/+ (P-LP) | Peru (1) |
| 71 | *MYBPC3* | | 1 | c.2864_2865delCT | [rs397515990](http://www.ncbi.nlm.nih.gov/snp/rs397515990) | Pathogenic | Pro955Argfs*95 | +/+ (P) | Argentina (1) |
| 72 | *MYBPC3* | | 1 | c.3257G>A | [rs779650200](http://www.ncbi.nlm.nih.gov/snp/rs779650200) | Pathogenic | Trp1086* | +/+ (P) | Colombia (1) |
| 73 | *MYBPC3* | | 1 | c.350dupC | N/A | Likely Pathogenic | Gly118Trpfs*9 | -/-** | Kazakhstan (1) |
| 74 | *MYBPC3* | | 1 | c.1310delT | [rs397515896](http://www.ncbi.nlm.nih.gov/snp/rs397515896) | Pathogenic | Val437Glyfs*13 | +/+ (P) | Argentina (1) |
| 75 | *MYBPC3* | | 1 | c.2943_2947delGACCA | [rs397515995](http://www.ncbi.nlm.nih.gov/snp/rs397515995) | Pathogenic | Gln981Hisfs*68 | +/+ (P) | Kazakhstan (1) |
| 76 | *MYBPC3* | | 1 | c.2309-2A>G | [rs111729952](http://www.ncbi.nlm.nih.gov/snp/rs111729952) | Pathogenic | splicing | +/+ (P) | Brazil (1) |
| 77 | *MYBPC3* | | 1 | c.3773T>G | [rs730880604](http://www.ncbi.nlm.nih.gov/snp/rs730880604) | Pathogenic | Leu1258* | +/+ (P) | Turkey (1) |
| 78 | *MYBPC3* | | 1 | c.2541C>G | [rs397515974](http://www.ncbi.nlm.nih.gov/snp/rs397515974) | Pathogenic | Tyr847* | +/+ (P) | Brazil (1) |
| 79 | *MYBPC3* | | 1 | c.1513_1515delAAG | [rs727504287](http://www.ncbi.nlm.nih.gov/snp/rs727504287) | Pathogenic | Lys505del | +/+ (LP-VUS) | Brazil (1) |
| 80 | *MYBPC3* | | 1 | c.529C>T | [rs193922385](http://www.ncbi.nlm.nih.gov/snp/rs193922385) | VUS | Arg177Cys | + (VUS)/+ (P) | Brazil (1) |
| 81 | *MYBPC3* | | 1 | c.3713T>C | [rs730880702](http://www.ncbi.nlm.nih.gov/snp/rs730880702) | Pathogenic | Leu1238Pro | +/+ (P) | Argentina (1) |
| 82 | *MYBPC3* | | 1 | c.913_914delTT | [rs397516080](http://www.ncbi.nlm.nih.gov/snp/rs397516080) | Pathogenic | Phe305ProfsTer27 | +/+ (P) | Argentina (1) |
| 83 | *MYH7* | | 14 | c.788T>C | [rs397516269](http://www.ncbi.nlm.nih.gov/snp/rs397516269) | Pathogenic | Ile263Thr | +/+ (P-LP) | Argentina (2), Brazil (9), Chile (2), Colombia (1) |
| 84 | *MYH7* | | 9 | c.2389G>A | [rs3218716](http://www.ncbi.nlm.nih.gov/snp/rs3218716) | Pathogenic | Ala797Thr | +/+ (P) | Argentina (1), Brazil (7), Colombia (1) |
| 85 | *MYH7* | | 9 | c.1988G>A | [rs371898076](http://www.ncbi.nlm.nih.gov/snp/rs371898076) | Pathogenic | Arg663His | +/+ (P) | Brazil (5), Colombia (2), Turkey (2) |
| 86 | *MYH7* | | 8 | c.2207T>C | [rs727503261](http://www.ncbi.nlm.nih.gov/snp/rs727503261) | Pathogenic | Ile736Thr | +/+ (P) | Argentina (2), Brazil (1), Colombia (2), Kazakhstan (3) |
| 87 | *MYH7* | | 7 | c.428G>A | [rs397516209](http://www.ncbi.nlm.nih.gov/snp/rs397516209) | Pathogenic | Arg143Gln | +/+ (LP-P) | Brazil (2), Kazakhstan (4), Peru (1) |
| 88 | *MYH7* | | 6 | c.1357C>T | [rs121913625](http://www.ncbi.nlm.nih.gov/snp/rs121913625) | Pathogenic | Arg453Cys | +/+ (P) | Algeria (1), Brazil (4), Kazakhstan (1) |
| 89 | *MYH7* | | 5 | c.1816G>A | [rs121913627](http://www.ncbi.nlm.nih.gov/snp/rs121913627) | Pathogenic | Val606Met | +/+ (P) | Algeria (3), Argentina (1), Colombia (1) |
| 90 | *MYH7* | | 5 | c.727C>T | [rs397516265](http://www.ncbi.nlm.nih.gov/snp/rs397516265) | Pathogenic | Arg243Cys | +/+ (LP-P) | Colombia (5) |
| 91 | *MYH7* | | 4 | c.2146G>A | [rs121913638](http://www.ncbi.nlm.nih.gov/snp/rs121913638) | Pathogenic | Gly716Arg | +/+ (P) | Brazil (2), Colombia (2) |
| 92 | *MYH7* | | 4 | c.2302G>A | [rs727503260](http://www.ncbi.nlm.nih.gov/snp/rs727503260) | Pathogenic | Gly768Arg | +/+ (P) | Colombia (3), Mexico (1) |
| 93 | *MYH7* | | 4 | c.2605C>T | [rs730880750](http://www.ncbi.nlm.nih.gov/snp/rs730880750) | Pathogenic | Arg869Cys | +/+ (P-LP) | Brazil (2), Colombia (1), Taiwan (1) |
| 94 | *MYH7* | | 4 | c.2167C>G | [rs121913630](http://www.ncbi.nlm.nih.gov/snp/rs121913630) | Pathogenic | Arg723Gly | +/+ (P) | Argentina (1), Brazil (3) |
| 95 | *MYH7* | | 4 | c.746G>A | [rs3218713](http://www.ncbi.nlm.nih.gov/snp/rs3218713) | Pathogenic | Arg249Gln | +/+ (P) | Argentina (3), Taiwan (1) |
| 96 | *MYH7* | | 3 | c.611G>A | [rs397516260](http://www.ncbi.nlm.nih.gov/snp/rs397516260) | Pathogenic | Arg204His | +/+ (P-LP-VUS) | Brazil (1), Kazakhstan (1), Taiwan (1) |
| 97 | *MYH7* | | 3 | c.1063G>A | [rs397516088](http://www.ncbi.nlm.nih.gov/snp/rs397516088) | Pathogenic | Ala355Thr | +/+ (P-LP) | Argentina (2), Hong Kong (1) |
| 98 | *MYH7* | | 3 | c.1208G>A | [rs121913624](http://www.ncbi.nlm.nih.gov/snp/rs121913624) | Pathogenic | Arg403Gln | +/+ (P) | Algeria (1), Colombia (1), Mexico (1) |
| 99 | *MYH7* | | 3 | c.2155C>T | [rs121913637](http://www.ncbi.nlm.nih.gov/snp/rs121913637) | Pathogenic | Arg719Trp | -/+ (P) | Argentina (1), Brazil (1), Colombia (1) |
| 100 | *MYH7* | | 3 | c.1436A>G | [rs727504236](http://www.ncbi.nlm.nih.gov/snp/rs727504236) | Pathogenic | Asn479Ser | +/+ (P-LP) | Brazil (3) |
| 101 | *MYH7* | | 3 | c.1987C>T | [rs397516127](http://www.ncbi.nlm.nih.gov/snp/rs397516127) | Pathogenic | Arg663Cys | +/+ (P) | Hong Kong (3) |
| 102 | *MYH7* | | 2 | c.1370T>C | [rs397516103](http://www.ncbi.nlm.nih.gov/snp/rs397516103) | Pathogenic | Ile457Thr | +/+ (LP) | Israel (1), Kazakhstan (1) |
| 103 | *MYH7* | | 2 | c.1331A>G | [rs730880159](http://www.ncbi.nlm.nih.gov/snp/rs730880159) | Pathogenic | Asn444Ser | +/+ (P-LP-VUS) | Algeria (2) |
| 104 | *MYH7* | | 2 | c.2770G>A | [rs121913628](http://www.ncbi.nlm.nih.gov/snp/rs121913628) | Pathogenic | Glu924Lys | +/+ (P) | Mexico (2) |
| 105 | *MYH7* | | 2 | c.1207C>T | [rs3218714](http://www.ncbi.nlm.nih.gov/snp/rs3218714) | Pathogenic | Arg403Trp | +/+ (P) | Argentina (2) |
| 106 | *MYH7* | | 2 | c.1324C>T | [rs148808089](http://www.ncbi.nlm.nih.gov/snp/rs148808089) | Pathogenic | Arg442Cys | +/+ (P-LP) | Argentina (2) |
| 107 | *MYH7* | | 2 | c.2156G>A | [rs121913641](http://www.ncbi.nlm.nih.gov/snp/rs121913641) | Pathogenic | Arg719Gln | +/+ (P) | Brazil (2) |
| 108 | *MYH7* | | 2 | c.2539_2541delAAG | [rs397516155](http://www.ncbi.nlm.nih.gov/snp/rs397516155) | Pathogenic | Lys847del | +/+ (LP) | Kazakhstan (1), Mexico (1) |
| 109 | *MYH7* | | 2 | c.958G>A | [rs376897125](http://www.ncbi.nlm.nih.gov/snp/rs376897125) | Pathogenic | Val320Met | +/+ (P-LP) | Brazil (2) |
| 110 | *MYH7* | | 2 | c.2221G>C | [rs121913632](http://www.ncbi.nlm.nih.gov/snp/rs121913632) | Pathogenic | Gly741Arg | +/+ (P-LP) | Brazil (1), Panama (1) |
| 111 | *MYH7* | | 2 | c.2609G>A | [rs36211715](http://www.ncbi.nlm.nih.gov/snp/rs36211715) | Pathogenic | Arg870His | +/+ (P) | Argentina (1), Brazil (1) |
| 112 | *MYH7* | | 2 | c.5134C>T | [rs121913650](http://www.ncbi.nlm.nih.gov/snp/rs121913650) | Pathogenic | Arg1712Trp | +/+ (P-LP) | Brazil (1), Kazakhstan (1) |
| 113 | *MYH7* | | 2 | c.2606G>A | [rs202141173](http://www.ncbi.nlm.nih.gov/snp/rs202141173) | Pathogenic | Arg869His | +/+ (P-LP) | Argentina (1), Kazakhstan (1) |
| 114 | *MYH7* | | 2 | c.2092G>T | N/A | Likely Pathogenic | Val698Leu | -/-** | Hong Kong (2) |
| 115 | *MYH7* | | 1 | c.506G>A | [rs397516237](http://www.ncbi.nlm.nih.gov/snp/rs397516237) | Likely Pathogenic | Arg169Lys | +/+ (LP) | Israel (1) |
| 116 | *MYH7* | | 1 | c.1750G>C | [rs121913626](http://www.ncbi.nlm.nih.gov/snp/rs121913626) | Pathogenic | Gly584Arg | +/+ (P-LP) | Brazil (1) |
| 117 | *MYH7* | | 1 | c.2791G>A | [rs1131691514](http://www.ncbi.nlm.nih.gov/snp/rs1131691514) | Pathogenic | Glu931Lys | +/+ (P-LP) | Colombia (1) |
| 118 | *MYH7* | | 1 | c.1615A>C | [rs730880930](http://www.ncbi.nlm.nih.gov/snp/rs730880930) | Pathogenic | Met539Leu | +/+ (LP) | Argentina (1) |
| 119 | *MYH7* | | 1 | c.2821C>T | N/A | Likely Pathogenic | Arg941Cys | +/+ (LP) | Brazil (1) |
| 120 | *MYH7* | | 1 | c.1750G>A | [rs121913626](http://www.ncbi.nlm.nih.gov/snp/rs121913626) | Pathogenic | Gly584Ser | +/+ (P-LP) | Brazil (1) |
| 121 | *MYH7* | | 1 | c.715G>A | [rs397516264](http://www.ncbi.nlm.nih.gov/snp/rs397516264) | Pathogenic | Asp239Asn | +/+ (P-LP) | Brazil (1) |
| 122 | *MYH7* | | 1 | c.5655G>A | [rs753392652](http://www.ncbi.nlm.nih.gov/snp/rs753392652) | Likely Pathogenic | Ala1885= | -/+ (P-LP) | Brazil (1) |
| 123 | *MYH7* | | 1 | c.2451C>A | [rs876661372](http://www.ncbi.nlm.nih.gov/snp/rs876661372) | Likely Pathogenic | Asn817Lys | + (VUS)/+ (VUS) | Brazil (1) |
| 124 | *MYH7* | | 1 | c.1013T>A | [rs397516087](http://www.ncbi.nlm.nih.gov/snp/rs397516087) | Likely Pathogenic | Val338Glu | + (VUS)/+ (VUS) | Brazil (1) |
| 125 | *MYH7* | | 1 | c.1479G>A | [rs730880876](http://www.ncbi.nlm.nih.gov/snp/rs730880876) | Pathogenic | Met493Ile | + /+ (LP-VUS) | Colombia (1) |
| 126 | *MYH7* | | 1 | c.1988G>A | [rs371898076](http://www.ncbi.nlm.nih.gov/snp/rs371898076) | Pathogenic | Arg663His) | +/+ (P) | Brazil (1) |
| 127 | *MYH7* | | 1 | c.745C>G | [rs730880852](http://www.ncbi.nlm.nih.gov/snp/rs730880852) | Likely Pathogenic | Arg249Gly | + (VUS)/+ (VUS-LP) | Kazakhstan (1) |
| 128 | *MYH7* | | 1 | c.1452G>T | N/A | Likely Pathogenic | Lys484Asn | -/+ (VUS) | Algeria (1) |
| 129 | *MYH7* | | 1 | c.4130C>T | [rs397516201](http://www.ncbi.nlm.nih.gov/snp/rs397516201) | Pathogenic | Thr1377Met | +/+ (P) | Colombia (1) |
| 130 | *MYH7* | | 1 | c.1331A>C | [rs730880159](http://www.ncbi.nlm.nih.gov/snp/rs730880159) | Pathogenic | Asn444Ser | + /+ (LP) | Algeria (1) |
| 131 | *MYH7* | | 1 | c.1477A>G | [rs730880875](http://www.ncbi.nlm.nih.gov/snp/rs730880875) | Pathogenic | Met493Val | + /+ (LP) | Brazil (1) |
| 132 | *MYH7* | | 1 | c.2713T>C | N/A | Likely Pathogenic | Cys905Arg | -/-** | Colombia (1) |
| 133 | *MYH7* | | 1 | c.438G>C | N/A | Pathogenic | Lys146Asn | + (VUS)/+ (LP) | Argentina (1) |
| 134 | *MYH7* | | 1 | c.4066G>A | [rs727503246](http://www.ncbi.nlm.nih.gov/snp/rs727503246) | Pathogenic | Glu1356Lys | +/+ (P-LP) | Colombia (1) |
| 135 | *MYH7* | | 1 | c.2761G>A | [rs730880759](http://www.ncbi.nlm.nih.gov/snp/rs730880759) | Pathogenic | Glu921Lys | +/+ (P-LP) | Argentina (1) |
| 136 | *MYH7* | | 1 | c.5342G>A | [rs397516246](http://www.ncbi.nlm.nih.gov/snp/rs397516246) | Likely Pathogenic | Arg1781His | +/+ (P-VUS) | Argentina (1) |
| 1370 | *MYH7* | | 1 | c.4259G>A | [rs397516207](http://www.ncbi.nlm.nih.gov/snp/rs397516207) | Pathogenic | Arg1420Gln | +/+ (P-LP) | Kazakhstan (1) |
| 138 | *MYH7* | | 1 | c.2189T>A | N/A | Pathogenic | Ile730Asn | +/+ (LP) | Argentina (1) |
| 139 | *MYH7* | | 1 | c.920C>G | N/A | Likely Pathogenic | Pro307Arg | +/- | Kazakhstan (1) |
| 140 | *MYH7* | | 1 | c.2770G>C | rs121913628 | Likely Pathogenic | Glu924Gln | +/+ (P) | Peru (1) |
| 141 | *MYH7* | | 1 | c.2332G>C | N/A | Likely Pathogenic | Asp778His | -/+ (LP) | Brazil (1) |
| 142 | *MYH7* | | 1 | c.3158G>A | [rs587782962](http://www.ncbi.nlm.nih.gov/snp/rs587782962) | Pathogenic | Arg1053Gln | +/+ (P) | Colombia (1) |
| 143 | *MYH7* | | 1 | c.2544G>C | [rs730880899](http://www.ncbi.nlm.nih.gov/snp/rs730880899) | Pathogenic | Glu848Asp | + (VUS) /+ (LP-VUS) | Brazil (1) |
| 144 | *TNNI3* | | 4 | c.422G>A | [rs397516347](http://www.ncbi.nlm.nih.gov/snp/rs397516347) | Pathogenic | Arg141Gln | +/+ (P-LP) | Chile (1), Colombia (3) |
| 145 | *TNNI3* | | 3 | c.470C>T | [rs397516353](http://www.ncbi.nlm.nih.gov/snp/rs397516353) | Pathogenic | Ala157Val | +/+ (P) | Colombia (3) |
| 146 | *TNNI3* | | 3 | c.484C>T | [rs368861241](http://www.ncbi.nlm.nih.gov/snp/rs368861241) | Pathogenic | Arg162Trp | +/+ (P-LP-VUS) | Brazil (2), Colombia (1) |
| 147 | *TNNI3* | | 2 | c.204delG | [rs727504872](http://www.ncbi.nlm.nih.gov/snp/rs727504872) | Pathogenic | Arg69Alafs*8 | +/+ (P-LP-VUS) | Algeria (2) |
| 148 | *TNNI3* | | 2 | c.557G>A | [rs397516357](http://www.ncbi.nlm.nih.gov/snp/rs397516357) | Pathogenic | Arg186Gln | +/+ (P) | Brazil (1), Colombia (1) |
| 149 | *TNNI3* | | 1 | c.370G>C | [rs727503506](http://www.ncbi.nlm.nih.gov/snp/rs727503506) | Likely Pathogenic | Glu124Gln | +/+ (LP-VUS) | Hong Kong (1) |
| 150 | *TNNI3* | | 1 | c.485G>A | [rs397516354](http://www.ncbi.nlm.nih.gov/snp/rs397516354) | Pathogenic | Arg162Gln | +/+ (P-LP) | Hong Kong (1) |
| 151 | *TNNI3* | | 1 | c.575G>A | [rs104894729](http://www.ncbi.nlm.nih.gov/snp/rs104894729) | Pathogenic | Arg192His | +/+ (P) | Peru (1) |
| 152 | *TNNI3* | | 1 | c.485G>C | [rs397516354](http://www.ncbi.nlm.nih.gov/snp/rs397516354) | Pathogenic | Arg162Pro | +/+ (P-LP) | Mexico (1) |
| 153 | *TNNI3* | | 1 | c.433C>T | [rs104894724](http://www.ncbi.nlm.nih.gov/snp/rs104894724) | Pathogenic | Arg145Trp | +/+ (P) | Turkey (1) |
| 154 | *TNNI3* | | 1 | c.610C>T | [rs727504243](http://www.ncbi.nlm.nih.gov/snp/rs727504243) | Pathogenic | Arg204Cys | +/+ (P-LP-VUS) | Brazil (1) |
| 155 | *TNNI3* | | 1 | c.526G>A | [rs727503501](http://www.ncbi.nlm.nih.gov/snp/rs727503501) | Likely Pathogenic | Val176Met | +/+ (LP-VUS) | Colombia (1) |
| 156 | *TNNI3* | | 1 | c.434G>A | [rs397516349](http://www.ncbi.nlm.nih.gov/snp/rs397516349) | Pathogenic | Arg145Gln | +/+ (P-LP) | UAE (1) |
| 157 | *TNNI3* | | 1 | c.336C>A | [rs559450042](http://www.ncbi.nlm.nih.gov/snp/rs559450042) | Likely Pathogenic | Tyr112* | -/-** | Algeria (1) |
| 158 | *TNNI3* | | 1 | c.586G>A | [rs104894727](http://www.ncbi.nlm.nih.gov/snp/rs104894727) | Pathogenic | Asp196Asn | +/+ (P-LP) | Israel (1) |
| 159 | *TNNT2* | | 3 | c.304C>T | [rs397516456](http://www.ncbi.nlm.nih.gov/snp/rs397516456) | Pathogenic | Arg102Trp | +/+ (P) | Argentina (1), Brazil (1), Mexico (1) |
| 160 | *TNNT2* | | 3 | c.833A>T | [rs863225119](http://www.ncbi.nlm.nih.gov/snp/rs863225119) | Pathogenic | Asn278Ile | +/+ (P-LP-VUS) | Argentina (2), Brazil (1) |
| 161 | *TNNT2* | | 2 | c.418C>T | [rs397516463](http://www.ncbi.nlm.nih.gov/snp/rs397516463) | Pathogenic | Arg140Cys | +/+ (P-LP) | Brazil (2) |
| 162 | *TNNT2* | | 2 | c.547C>T | [rs727503512](http://www.ncbi.nlm.nih.gov/snp/rs727503512) | Pathogenic | Arg183Trp | +/+ (P) | Turkey (2) |
| 163 | *TNNT2* | | 1 | c.517_519delGAG | [rs397516470](http://www.ncbi.nlm.nih.gov/snp/rs397516470) | Pathogenic | Glu173del | +/+ (P-LP) | UAE (1) |
| 164 | *TNNT2* | | 1 | c.305G>A | [rs121964856](http://www.ncbi.nlm.nih.gov/snp/rs121964856) | Pathogenic | Arg102Gln | +/+ (P-LP) | Hong Kong (1) |
| 165 | *TNNT2* | | 1 | c.411+1G>A | [rs766666484](http://www.ncbi.nlm.nih.gov/snp/rs766666484) | Likely Pathogenic | splicing | -/+ (VUS) | Brazil (1) |
| 166 | *TNNT2* | | 1 | c.886C>T | [rs367785431](http://www.ncbi.nlm.nih.gov/snp/rs367785431) | Pathogenic | Arg296Cys | +/+ (P-LP-VUS) | Hong Kong (1) |
| 167 | *TNNT2* | | 1 | c.311G>A | [rs397516457](http://www.ncbi.nlm.nih.gov/snp/rs397516457) | Pathogenic | Arg104His | +/+ (P) | Algeria (1) |
| 168 | *TPM1* | | 3 | c.644C>T | [rs199476316](http://www.ncbi.nlm.nih.gov/snp/rs199476316) | Pathogenic | Ser215Leu | +/+ (P-LP) | Argentina (2), Brazil (1) |
| 169 | *TPM1* | | 3 | c.842T>C | [rs199476321](http://www.ncbi.nlm.nih.gov/snp/rs199476321) | Pathogenic | Met281Thr | +/+ (P-LP) | Saudi Arabia (1), UAE (2) |
| 170 | *TPM1* | | 2 | c.523G>A | [rs104894503](http://www.ncbi.nlm.nih.gov/snp/rs104894503) | Pathogenic | Asp175Asn | +/+ (P) | Colombia (2) |
| 171 | *TPM1* | | 1 | c.574G>A | [rs199476315](http://www.ncbi.nlm.nih.gov/snp/rs199476315) | Pathogenic | Glu192Lys | +/+ (P) | Israel (1) |
| 172 | *MYL2* | | 1 | c.484G>T | N/A | Likely Pathogenic | Gly162* | -/+ (VUS) | Argentina (1) |
| 173 | *MYL2* | | 1 | c.484G>A | [rs199474814](http://www.ncbi.nlm.nih.gov/snp/rs199474814) | Pathogenic | Gly162Arg | +/+ (P-LP) | Brazil (1) |
| 174 | *MYL2* | | 1 | c.173G>A | [rs104894369](http://www.ncbi.nlm.nih.gov/snp/rs104894369) | Pathogenic | Arg58Gln | +/+ (P) | Peru (1) |
| 175 | *MYL3* | | 1 | c.427G>A | [rs104893750](http://www.ncbi.nlm.nih.gov/snp/rs104893750) | Pathogenic | Glu143Lys | +/+ (P-LP) | Guatemala (1) |
| 176 | *CSRP3* | | 1 | c.10_11delTG | [rs1554968150](http://www.ncbi.nlm.nih.gov/snp/rs1554968150) | Pathogenic | Trp4Glyfs*10 | -/+ (VUS) | Brazil (1) |
| 177 | *CSRP3* | | 1 | c.362delC | [rs1565050709](http://www.ncbi.nlm.nih.gov/snp/rs1565050709) | Pathogenic | Pro121Leufs*87 | -/+ (LP-VUS) | Brazil (1) |
| 178 | *CSRP3* | | 1 | c.128delC | [rs1187710437](http://www.ncbi.nlm.nih.gov/snp/rs1187710437) | Pathogenic | Ala43Valfs*165 | +/+ (LP-VUS) | Argentina (1) |
| 179 | *TNNC1* | | 7 | c.23C>T | [rs267607125](http://www.ncbi.nlm.nih.gov/snp/rs267607125) | Pathogenic | Ala8Val | +/+ (P-LP) | Argentina (5), Colombia (1), Peru (1) |
|  | Total | | 380* |  |  |  |  |  |  |
|  | | *Among 380 patients, 3 had double variants.  **A total of 16 novel variants were identified (13 in *MYBPC3,* 2 in *MYH7,* 1 in *TNNI3*). | | | | | | | |
