# Supplementary material for: Uncovering the gene variants in a global cohort of patients with unexplained increased left ventricular wall thickness using next-generation sequencing
Source: BMC Cardiovasc Disord. 2026 Apr 17;26:463. doi: 10.1186/s12872-026-05834-5 (PMC13231718; doi:10.1186/s12872-026-05834-5)
Supplement: Supplementary file 2 — Supplementary Material 2. [file 12872_2026_5834_MOESM2_ESM.docx]

**Supplementary Table S2:** Classifications of pathogenicity and nucleotide/amino acid changes for all hypertrophic cardiomyopathy phenocopy positive variants.

| **Sl. No.** | **Gene** | **Number of cases** | **Nucleotide change** | **Reference SNP (rs) No** | **ACMGclassification** | **Amino acid change** | **Novel HGMD/ ClinVar** | **Countries** |
| --- | --- | --- | --- | --- | --- | --- | --- | --- |
| 1 | *TTR* | 23 | c.424G>A | [rs76992529](http://www.ncbi.nlm.nih.gov/snp/rs76992529) | Likely Pathogenic | Val142Ile | +/+ (P) | Brazil (14), Colombia (8), Peru (1) |
| 2 | *TTR* | 5 | c.148G>A | [rs28933979](http://www.ncbi.nlm.nih.gov/snp/rs28933979) | Pathogenic | Val50Met | +/+ (P) | Brazil (3), Colombia (2) |
| 3 | *TTR* | 2 | c.209G>A | [rs121918080](http://www.ncbi.nlm.nih.gov/snp/rs121918080) | Likely Pathogenic | Ser70Asn | +/+ (LP-VUS) | Brazil (2) |
| 4 | *TTR* | 1 | c.239C>T | [rs1254341785](http://www.ncbi.nlm.nih.gov/snp/rs1254341785) | Pathogenic | Thr80Ile | +/+ (P-LP) | Saudi Arabia (1) |
| 5 | *TTR* | 1 | c.258A>T | N/A | Pathogenic | Glu86Asp | +/+ (LP) | Taiwan (1) |
| 6 | *GLA* | 3 | c.1066C>T | [rs104894827](http://www.ncbi.nlm.nih.gov/snp/rs104894827) | Pathogenic | Arg356Trp | +/+ (P) | Colombia (3) |
| 7 | *GLA* | 3 | c.640-801G>A | [rs199473684](http://www.ncbi.nlm.nih.gov/snp/rs199473684) | Pathogenic | Splicing | +/+ (P) | Taiwan (2), Hong Kong (1) |
| 8 | *GLA* | 3 | c.520T>G | [rs181562693](http://www.ncbi.nlm.nih.gov/snp/rs181562693) | Likely Pathogenic | Cys174Gly | +/- | Argentina (3) |
| 9 | *GLA* | 2 | c.644A>G | [rs28935197](http://www.ncbi.nlm.nih.gov/snp/rs28935197) | Pathogenic | Asn215Ser | +/+ (P) | Brazil (1), Argentina (1) |
| 10 | *GLA* | 2 | c.870G>C | [rs869312438](http://www.ncbi.nlm.nih.gov/snp/rs869312438) | Pathogenic | Met290Ile | +/+ (P) | Brazil (2) |
| 11 | *GLA* | 1 | c.1088G>A | [rs111422676](http://www.ncbi.nlm.nih.gov/snp/rs111422676) | Pathogenic | Arg363His | +/+ (P) | Mexico (1) |
| 12 | *GLA* | 1 | c.419A>C | [rs150228150](http://www.ncbi.nlm.nih.gov/snp/rs150228150) | Likely Pathogenic | Lys140Thr | +/+ (VUS) | Colombia (1) |
| 13 | *GLA* | 1 | c.41T>C | [rs730880455](http://www.ncbi.nlm.nih.gov/snp/rs730880455) | Pathogenic | Arg118Cys | +/+ (LP) | Hong Kong (1) |
| 14 | *GLA* | 1 | c.50_54dup GCTTC | N/A | Likely Pathogenic | Leu19Alafs*104 | -/-* | Argentina (1) |
| 15 | *GLA* | 1 | c.525C>A | [rs782722844](http://www.ncbi.nlm.nih.gov/snp/rs782722844) | Likely Pathogenic | Asp175Glu | --/+ (VUS) | Algeria (1) |
| 16 | *LAMP2* | 1 | c.138G>A | [rs1271031981](http://www.ncbi.nlm.nih.gov/snp/rs1271031981) | Pathogenic | Trp46* | +/+ (P) | Brazil (1) |
| 17 | *LAMP2* | 1 | c.973dupC | [rs1556092459](http://www.ncbi.nlm.nih.gov/snp/rs1556092459) | Pathogenic | Leu325Profs*25 | +/+ (P) | Mexico (1) |
| 18 | *LAMP2* | 1 | c.190_191delGT | [rs1569371330](http://www.ncbi.nlm.nih.gov/snp/rs1569371330) | Pathogenic | Val64AsnfsTer11 | +/+ (P) | Brazil (1) |
| 19 | *LAMP2* | 1 | c.869dupA | N/A | Likely Pathogenic | Asn290Lysfs*2 | - /-* | Colombia (1) |
| 20 | *PTPN11* | 4 | c.836A>G | [rs121918456](http://www.ncbi.nlm.nih.gov/snp/rs121918456) | Pathogenic | Tyr279Cys | +/+ (P) | Algeria (1), Brazil (2), Kazakhstan (1) |
| 21 | *PTPN11* | 3 | c.1415C>T | [rs121918457](http://www.ncbi.nlm.nih.gov/snp/rs121918457) | Pathogenic | Thr472Met | +/+ (P) | Brazil (2), Colombia (1) |
| 22 | *PTPN11* | 1 | c.1528C>G | [rs397507549](http://www.ncbi.nlm.nih.gov/snp/rs397507549) | Pathogenic | Gln510Glu | +/+ (P) | Algeria (1) |
| 23 | *PTPN11* | 1 | c.922A>G | [rs28933386](http://www.ncbi.nlm.nih.gov/snp/rs28933386) | Pathogenic | Asn308Asp | +/+ (P) | Brazil (1) |
| 24 | *PRKAG2* | 2 | c.905G>A | [rs121908987](http://www.ncbi.nlm.nih.gov/snp/rs121908987) | Pathogenic | Arg302Gln | +/+ (P) | Brazil (1), Colombia (1) |
| 25 | *PRKAG2* | 1 | c.1199C>A | [rs28938173](http://www.ncbi.nlm.nih.gov/snp/rs28938173) | Likely Pathogenic | Thr400Asn | +/+ (P-LP) | Brazil (1) |
| 26 | *DES* | 2 | c.1360C>T | [rs267607490](http://www.ncbi.nlm.nih.gov/snp/rs267607490) | Pathogenic | Arg454Trp | +/+ (P-LP) | South Africa (2) |
| 27 | *DES* | 1 | c.735G>C | [rs267607486](http://www.ncbi.nlm.nih.gov/snp/rs267607486) | Likely Pathogenic | Glu245Asp | -/+ (P-LP) | Mexico (1) |
| 28 | *FLNC* | 1 | c.3562delC | N/A | Likely Pathogenic | Val1188* | -/-* | Hong Kong (1) |
| 29 | *FLNC* | 1 | c.2813delG | N/A | Likely Pathogenic | Gly938Alafs*5 | -/-* | Brazil (1) |
| 30 | *FLNC* | 1 | c.6976C>T | [rs748416758](http://www.ncbi.nlm.nih.gov/snp/rs748416758) | Pathogenic | Arg2326* | +/+ (P) | Brazil (1) |
| 31 | *FLNC* | 1 | c.1156G>T | N/A | Pathogenic | Glu386* | -/+ (P) | Colombia (1) |
|  | Total | 73 |  |  |  |  |  |  |

* A total of 4 novel variants were identified (2 in *FLNC,* 1 in *LAMP2,* 1 in *GLA*).
